# Human Schwann‐like cells derived from adipose‐derived mesenchymal stem cells rapidly de‐differentiate in the absence of stimulating medium

**DOI:** 10.1111/ejn.13055

**Published:** 2015-09-18

**Authors:** Alessandro Faroni, Richard J. P. Smith, Li Lu, Adam J. Reid

**Affiliations:** ^1^ Blond McIndoe Laboratories, Stopford BuildingCentre for Tissue Injury and RepairInstitute of Inflammation and RepairUniversity of ManchesterOxford RoadManchesterM13 9PTUK; ^2^ Department of PharmacologySchool of Basic MedicineLanzhou UniversityLanzhouChina; ^3^ Department of Plastic Surgery & BurnsUniversity Hospital of South ManchesterManchesterUK

**Keywords:** cell differentiation, growth factors, peripheral nerve injury, peripheral nerve regeneration

## Abstract

Finding a viable cell‐based therapy to address peripheral nerve injury holds promise for enhancing the currently suboptimal microsurgical approaches to peripheral nerve repair. Autologous nerve grafting is the current gold standard for surgical repair of nerve gaps; however, this causes donor nerve morbidity in the patient, and the results remain unsatisfactory. Transplanting autologous Schwann cells (SCs) results in similar morbidity, as well as limited cell numbers and restricted potential for expansion *in vitro*. Adipose‐derived stem cells (ASCs), ‘differentiated’ towards an SC‐like phenotype *in vitro* (dASCs), have been presented as an alternative to SC therapies. The differentiation protocol stimulates ASCs to mimic the SC phenotype; however, the efficacy of dASCs in nerve repair is not yet convincing, and the practicality of the SC‐like phenotype is unproven. Here, we examined the stability of dASCs by withdrawing differentiation medium for 72 h after the full 18‐day differentiation protocol, and measuring changes in morphology, gene expression, and protein levels. Withdrawal of differentiation medium from dASCs resulted in a rapid reversion to stem cell‐like characteristics. Quantitative real‐time polymerase chain reaction and enzyme‐linked immunosorbent assay analyses demonstrated a significant reduction in gene and protein expression of growth factors that were expressed at high levels following ‘differentiation’. Therefore, we question the relevance of differentiation to an SC‐like phenotype, as withdrawal of differentiation medium, a model of transplantation into an injured nerve, results in rapid reversion of the dASC phenotype to stem cell‐like characteristics. Further investigation into the differentiation process and the response of dASCs to an injured environment must be undertaken prior to the use of dASCs in peripheral nerve repair therapies.

## Introduction

Cell‐based therapies are poised to provide the most promising surgical intervention for improved functional outcomes in peripheral nerve injuries, especially in the presence of a peripheral nerve gap (Faroni *et al*., 2015). The current clinical treatment of autologous nerve grafting is limited by a microsurgical approach alone, which cannot overcome the complex neurobiology of peripheral nerve gap injury, including a lack of Schwann cells (SCs) or the denervated SC phenotype in the distal stump. The use of SCs as a therapy has been explored, with experimental success (Guenard *et al*., 1992; Mosahebi *et al*., 2001); however, the clinical use of SCs is problematic, owing to the need to sacrifice a functional nerve, and their limited expansion capacity (Tohill & Terenghi, 2004). Attention has therefore shifted to stem cell technology.

Human stem cells that have ‘differentiated’ towards a SC phenotype have demonstrated great experimental promise in several models of peripheral nerve regeneration. Human embryonic stem cells and induced pluripotent stem cells can generate SC‐like cells, which have functional behaviour in both *in vitro* and *in vivo* models (Lee *et al*., 2010; Ziegler *et al*., 2011; Ikeda *et al*., 2014). However, currently, ethical considerations and the risks of teratoma formation prevent the clinical use of these cell types. As an alternative, adult stem cells, including skin‐derived precursors and mesenchymal stem cells (MSCs), are subject to none of these concerns, and have multipotent properties, including ‘transdifferentiation’ across lineage towards a SC phenotype (Faroni *et al*., 2015; Sparling *et al*., 2015).

MSCs sourced from human bone marrow have demonstrated potential (Shimizu *et al*., 2007); however, adipose‐derived stem cells (ASCs) have emerged as a more likely source for clinical use, owing to the ease of harvesting, a 500‐fold greater yield, greater proliferation rates, and their immune‐privileged potential (Zuk *et al*., 2001; Kern *et al*., 2006). Rat ASCs were first demonstrated to differentiate into SCs by use of the Dezawa protocol for bone marrow MSCs (Dezawa *et al*., 2001; Kingham *et al*., 2007). Rat SC‐like ASCs have been extensively characterized for the expression of neuroglial markers, neurotrophic factors, and neurotransmitters and their receptors, as well as for their regenerative and myelinating potential, by the use of *in vitro* and *in vivo* models of nerve injury (di Summa *et al*., 2010; Kaewkhaw *et al*., 2011; Faroni *et al*., 2012, 2013a; Tomita *et al*., 2012). Subsequently, human ASCs have been stimulated with the same protocol to establish a SC‐like phenotype, which increases the neurotrophic and angiogenic potential of the cells (Tomita *et al*., 2013; Kingham *et al*., 2014). Nevertheless, there is a limited amount of information on the characteristics of human SC‐like cells and on how they compare with rat SC‐like cells in terms of phenotype and regenerative potential. In *in vivo* models of peripheral nerve gap repair, however, there was no demonstrable improvement in the regenerative effect of stimulating human ASCs (Kingham *et al*., 2014), although the stimulated cells do appear to have functional roles in myelinating the regenerating nerve in a similar model (Tomita *et al*., 2013).

It seems more likely that these cells are stimulated while in a permissive environment rather than truly transdifferentiated. For the clinical implementation of stem cell therapy for peripheral nerve repair, it is important to understand what becomes of the SC‐like phenotype following the *in vitro* ‘differentiation’ protocol. In this study, we demonstrate the effects of the protocol and subsequent withdrawal of the stimulating medium on human ASC morphology, proliferation, and gene and protein expression of key factors associated with SC function.

## Materials and methods

### Human adipose stem cell harvesting and culture

Samples of human subcutaneous abdominal adipose tissue were taken from four consenting patients undergoing reconstructive surgery at University Hospital South Manchester, UK. All patients were female, healthy, and aged 44–64 years. All procedures were approved by the National Research Ethics Committee, UK (NRES 13/SC/0499), and conformed with the World Medical Association Declaration of Helsinki. ASCs were isolated as previously described, with minor modifications (Kingham *et al*., 2007). Briefly, the adipose tissue was minced with a razor blade, and enzymatically dissociated with 0.2% (w/v) collagenase (Life Technologies, Paisley, UK) at 37 °C for 60 min.

The resulting digested tissue was filtered through a vacuum‐assisted 100‐μm nylon mesh (Merck Millipore UK, Watford, UK), and an equal volume of stem cell growth medium was added: α‐minimum essential Eagle's medium (αMEM) (Sigma‐Aldrich, Poole, UK), 10% (v/v) fetal bovine serum (FBS) (LabTech, Uckfield, UK), 2 mm l‐glutamine (GE Healthcare UK, Little Chalfont, UK), and 1% (v/v) penicillin–streptomycin solution. The tubes were centrifuged at 300 ***g*** for 10 min, the resulting pellet [the stromal vascular fraction (SVF)] was resuspended in 1 mL of Red Blood Cell Lysis Buffer (Sigma‐Aldrich) for 1 min, and 20 mL of αMEM was added to arrest lysis. The mixture was centrifuged at 300 ***g*** for 10 min, and the resulting pellet was either resuspended in αMEM and plated in T75 flasks for cell culture, or resuspended in flow cytometry buffer for characterization by flow cytometry (see below). Cultured cells were maintained in T75 flasks at 37 °C and 5% CO_2_, with three medium changes every week, and split when subconfluent.

### Stem cell characterization and assessments of multipotency

The characterization of surface marker expression on ASCs was carried out by flow cytometric analysis on SVF cells before plastic adherence, with anti‐human antibodies [MSC Phenotyping Cocktail (Miltenyi Biotec, Woking, UK; 130‐095‐198), CD271–allophycocyanin (APC) (Miltenyi Biotec; 130‐091‐884), and CD34–fluorescein isothiocyanate (FITC) (Miltenyi Biotec; 130‐098‐142)]. Immediately after separation from adipose tissue, the SVF cells were counted (Scepter 2.0 automated cell counter; Merck Millipore UK), and resuspended in 100 μL of flow cytometry buffer [0.5% bovine serum albumin (Sigma‐Aldrich) and 2 mm EDTA (Sigma‐Aldrich) in phosphate‐buffered saline (PBS) (Sigma Aldrich)], with 10 μL of antibody per 1 × 10^6^ cells. The mixture was incubated for 10 min in the dark at 4 °C. The cells were washed with 1 mL of flow cytometry buffer, and centrifuged at 300 ***g*** for 10 min. The cell pellet was resuspended in flow cytometry buffer and analysed in a Cyan ADP flow cytometer (Beckman Coulter, High Wycombe, UK). Appropriate isotype controls were used for every fluorophore [MSC Phenotyping Kit Isotypes (Miltenyi Biotec; 130‐095‐198), IgG_1_–APC (Miltenyi Biotec; 130‐099‐208), and IgG_2a_–FITC (Miltenyi Biotec; 130‐098‐877)]. Data were analysed with flowjo v10 (FlowJo LLC, Ashland, OR, USA).

To confirm multipotency, passage 1–2 ASCs were cultured in T75 flasks until they were confluent, and then plated in six‐well plates for chondrogenesis, adipogenesis, and osteogenesis. Induction media were changed every other day, and, for adipogenesis, a maintenance medium was required in place of the induction medium once weekly. The chondrogenic induction medium was: high‐glucose Dulbecco's modified Eagle's medium (DMEM) (Sigma‐Aldrich) plus 10% (v/v) FBS plus 1% (v/v) penicillin–streptomycin, containing 0.1 μm dexamethasone (Sigma‐Aldrich), 50 μg/mL ascorbate (Sigma‐Aldrich), 1% (v/v) ITS‐Premix (BD Biosciences, Oxford, UK), 40 μg/mL proline (Sigma‐Aldrich), and 50 μg/mL transforming growth factor‐β (R&D Systems, Minneapolis, MN, USA). The adipogenic induction medium was: low‐glucose DMEM (Sigma‐Aldrich) plus 10% FBS plus 1% penicillin–streptomycin, containing 1 μm dexamethasone, 10 μg/mL insulin (Sigma‐Aldrich), 0.5 mm 3‐isobutyl‐1‐methylxanthine (Sigma‐Aldrich) and 100 μm indomethacin (Sigma‐Aldrich). The adipogenic maintenance medium was: low‐glucose DMEM plus 10% FBS plus 1% penicillin–streptomycin, containing 10 μg/mL insulin. The osteogenic differentiation medium was: αMEM plus 10% FBS plus 1% penicillin–streptomycin, containing 0.1 μm dexamethasone, 100 μg/mL ascorbate, and 10 mm β‐glycerolphosphate (Sigma‐Aldrich). After 21 days of differentiation, cells were fixed with either 4% paraformaldehyde for 30 min or 10% formalin for 60 min, washed in PBS, and stained with the appropriate solutions: 0.5% (w/v) Toluidine Blue solution for the staining of proteoglycans produced by ASC‐derived chondrocytes, 0.2% (v/w) Oil Red O for the staining of fat droplets produced by ASC‐derived adipocytes, and 2% (v/w) Alizarin Red S for calcium deposits accumulated by ASC‐derived osteocytes (all Sigma‐Aldrich). Each staining was performed on unstimulated cultures as negative controls. Images were acquired with an Olympus IX51 inverted microscope (Olympus, Southend‐on‐Sea, UK).

### Differentiation to a Schwann‐like phenotype

Human undifferentiated ASCs (uASCs) were differentiated into Schwann‐like cells [SC‐like adipose‐derived stem cells (dASCs)] by use of a previously established protocol (Kingham *et al*., 2007, 2014). Passage 2 uASCs at 30% confluence were treated with 1 mm β‐mercaptoethanol (Sigma‐Aldrich) for 24 h, and this was followed by pre‐conditioning in 35 ng/mL all‐*trans*‐retinoic acid (Sigma‐Aldrich) for 72 h, before differentiation was started. The differentiation cocktail consisted of stem cell growth medium supplemented with 5 ng/mL platelet‐derived growth factor (Peprotech EC, London, UK), 10 ng/mL basic fibroblast growth factor (Peprotech EC), 14 mm forskolin (Sigma‐Aldrich), and 192 ng/mL glial growth factor‐2 (GGF‐2) (Acorda Therapeutics, Ardsley, NY, USA). Cells were split and replated (2 × 10^5^ cells in a 75‐cm^2^ flask) at day 4 and day 10 following growth factor induction, and RNA was collected 14 days into the differentiation protocol, for assessment of the differentiated phenotype. For all of the remaining experiments, passage 2–4 uASCs and passage 5–10 dASCs, which underwent at least 14 days of growth factor stimulation, were used.

### Cell size and morphology assessment

For assessment of the cell size, subconfluent uASCs and dASCs were trypsinized, and counted with a Scepter 2.0 automated cell counter (Merk Millipore UK). The cell count files were analysed with scepter software pro (v2.1; Merk Millipore UK) to obtain the mean cell diameter for each cell population.

For determination of cell morphology before and after glial differentiation, uASCs and dASCs from each patient (*n* = 4) were plated in eight‐well multichamber slides (Corning, Tewksbury, MA, USA) at a density of 5 × 10^4^ cells per well (four wells for each cell type/condition). After 24 h, cells were fixed for 20 min at room temperature with 4% (w/v) paraformaldehyde in PBS (Sigma‐Aldrich), permeabilized for 30 min with 0.2% (v/v) Triton‐X in PBS (Sigma‐Aldrich), and stained for 20 min at room temperature with Alexa 488‐conjugated phalloidin (1 : 40; Life Technologies) diluted in 1% (w/v) bovine serum albumin in PBS. Following further PBS washes, slides were mounted with aqueous Vectashield mounting medium with 4′,6‐diamidino‐2‐phenylindole (Vector Laboratories, Peterborough, UK), and fluorescent images were captured with an Olympus BX‐60 wide‐field microscope. Images were blindly analysed with image j (v1.47f; National Institutes of Health, Bethesda, MD, USA) to measure cell length (longest) and width (shortest); the aspect ratio was calculated as length/width for each cell measured. Fifteen to 20 cells were measured for each picture, and 12 pictures were taken for each experimental group (uASC/dASC) and for each patient (*n* = 4). Data were expressed as aspect ratio ± SE of the mean.

### Assessment of cell proliferation

To assess cell proliferation, uASCs and dASCs from four different patients were plated in 24‐well plates (Corning) at a density of 1 × 10^4^ cells per well in triplicate and for three time points. At days 2, 4 and 7 after plating, the medium was aspirated, and cells were then washed with PBS and incubated in 20% (v/v) CellTiter 96 AQueous One Solution Cell Proliferation Assay (Promega, Southampton, UK), diluted in phenol‐free DMEM (Sigma‐Aldrich). Following 90 min of incubation at 37 °C in the dark, the absorbance at 490 nm was recorded with an Asys UVM‐340 microplate reader/spectrophotometer (Biochrom, Cambridge, UK). Data were expressed as absorbance at 490 nm ± SE of the mean (*n* = 4).

### Withdrawal of the differentiation medium

To study the changes in dASC morphology and phenotype following the withdrawal of the differentiation medium, 1 × 10^5^ dASCs from each patient were plated in six‐well plates in triplicate for each experimental condition, in the medium supplemented with the differentiation cocktail. Following overnight incubation at 37 °C in 5% CO_2_, the medium was aspirated, and cells were washed with Hank's Balanced Salt Solution (Sigma‐Aldrich) to remove any remaining growth factors. Cells were then treated either with differentiation medium [dASCs maintained in differentiation medium, i.e. dASCs(+)] or with stem cell medium depleted of all growth factors and forskolin [dASCs deprived of differentiation medium, i.e. dASCs(−)] for 72 h. At the end of the incubation, supernatants were collected and snap‐frozen for enzyme‐linked immunosorbent assays (ELISAs). For cell size assessment, cells were trypsinized and analysed as described in the previous section; for the gene expression studies, cells were washed with ice‐cold PBS, collected in RNAprotect Cell reagent (Qiagen, Manchester, UK), and frozen until further analysis. For measurement of the aspect ratio, 2 × 10^4^ dASCs from each patient were seeded in 12‐well plates, and, following the growth factor withdrawal protocol, cells were fixed with paraformaldehyde and processed as in ‘Cell size and morphology assessment’.

### Quantitative real‐time polymerase chain reaction (qRT‐PCR)

For gene expression studies, RNA was extracted with the RNeasy Plus Mini Kit (Qiagen), according to the manufacturer's instructions. The RNA concentration was determined by spectrophotometric analysis with a NanoDrop ND‐100 (Thermo Fisher Scientific, Waltham, MA, USA), and 1 μg of each sample was reverse transcribed by use of the RT^2^ First Strand Kit (Qiagen), according to the manufacturer's protocol. Both RNA extraction and cDNA synthesis included DNA elimination steps to ensure the absence of downstream genomic DNA amplification. qRT‐PCR was performed with RT^2^ SYBR Green qPCR Mastermix (Qiagen) and a Corbett Rotor Gene 6000 (Qiagen), with the following protocol: hot start for 10 min at 95 °C, followed by 40 cycles of 15 s at 95 °C, annealing for 30 s at 55 °C, and extension for 30 s at 72 °C. To verify the specificity of the reactions, a melting curve was obtained with the following protocol: 95 °C for 1 min, 65 °C for 2 min, and a gradual temperature increase from 65 °C to 95 °C (2 °C/min). All of the primers for the genes of interest and for the housekeeping gene were laboratory‐verified RT^2^ qPCR Primer Assay primers (Qiagen), and are listed in Table 1. Data were normalized for the housekeeping gene, and the ΔΔCt method was used to determine the fold changes in gene expression, as compared with either uASCs (differentiation) or dASCs(+) (withdrawal) as controls.

**Table 1:** Primers used for qRT‐PCR analyses

Gene	Refseq accession no.	Catalogue no.	Band size (bp)
NGF	NM_002506	PPH00205F‐200	143
BDNF	NM_001709	PPH00569F‐200	86
NT‐3	NM_002527	PPH00687A‐200	151
GDNF	NM_000514	PPH01120B‐200	78
NRG1	NM_013957	PPH01151F‐200	100
TrkA	NM_002529.3	PPH01553A‐200	164
TrkB	NM_006180.3	PPH01552B‐200	136
TrkC	NM_002530.3	PPH01551A‐200	173
Ret	NM_020630.4	PPH00102F‐200	56
ErbB‐3	NM_001982	PPH00463B‐200	91
Nestin	NM_006617	PPH02388A‐200	161
Vimentin	NM_003380.3	PPH00417F‐200	92
Actin	NM_001101.3	PH00073G‐200	174
18S	X03205	PPH05666E‐200	100

All primers supplied by Qiagen as part of its RT^2^ qPCR primer assay kit.

### Elisa

To assess the levels of neurotrophic factors secreted by the cells, the supernatants collected in the sections above were analysed with ELISA kits for brain‐derived neurotrophic factor (BDNF), nerve growth factor (NGF), and glial‐derived neurotrophic factor (GDNF) (RayBiotech, Norcross, GA, USA), according to the manufacturer's instructions. For GDNF and NGF, before performance of the assays, supernatants were concentrated with Amicon Ultra‐4 Centifugal Filter Concentrator units (Merck Millipore), by centrifugation for 40 min at 3100 ***g***, which gave 10‐fold concentration. Each sample was assayed in triplicate, and the final absorbance was determined at 450 nm with an Asys UVM‐340 microplate spectrophotometer (Biochrom). The concentration values of secreted neurotrophic factors were extrapolated from standard curves produced from recombinant proteins provided in the kits.

### Statistical analysis

Statistical significance for the morphological, gene expression and protein level studies was evaluated by the use of graphpad prism 6.0f (Graphpad Software, La Jolla, CA, USA), with paired or unpaired two‐tailed Student's *t*‐tests; for the proliferation studies, a two‐way anova followed by Sidak's multiple comparison test was performed. Levels of significance were expressed as *P*‐values.

## Results

### Human adipose‐derived cell characterization and multipotency

**Figure 1:**
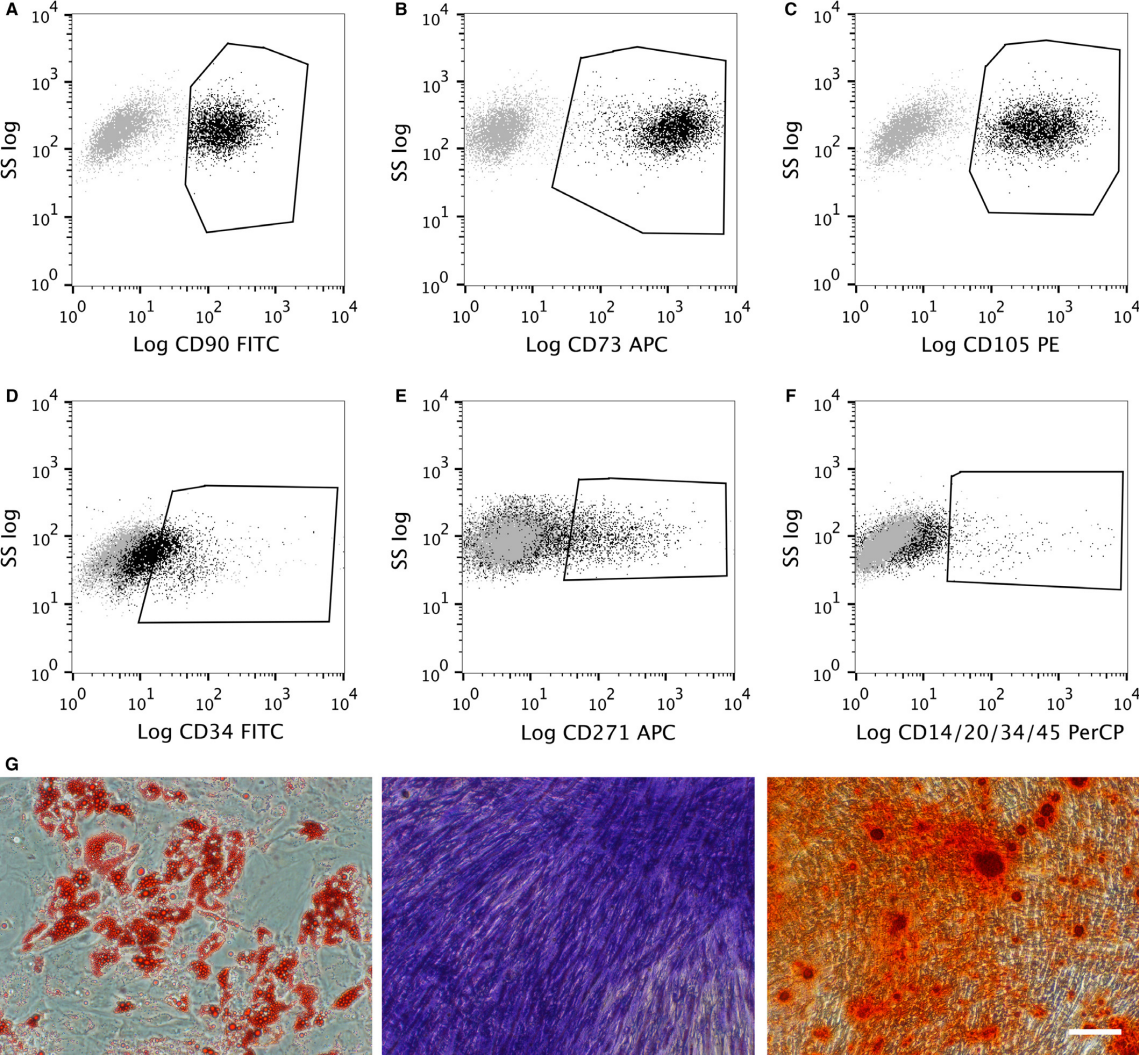
ASC characterization and evaluation of multipotency. (A–F) Analysis of surface marker expression on uASCs from the SVF by flow cytometry. Grey dots represent cells stained with isotype controls; black dots represent cells stained with fluorescent antibodies. Fluorescence is plotted against the log of side scatter (SS). (A–C) uASCs stained positively for CD90, CD73, and CD105. (D and E) Subpopulations of cell staining positively for CD34 and CD271 were observed. (F) uASCs stained mostly negatively for CD14/CD20/CD34/CD45. (G) Results of 21‐day trilineage differentiation of uASCs. The first panel shows the results of adipocytic differentiation with fat droplets stained with Oil Red O, followed by chondrogenic differentiation showing proteoglycans stained with Toluidine Blue, and finally osteogenic differentiation with calcium deposits stained with Alizarin Red S. Scale bar: 100 μm.

To determine the phenotype of freshly isolated uASCs, cells were stained with fluorescent antibodies for cell surface markers and analysed with flow cytometry (Fig. 1A–F). uASCs stained positively for the typical MSC markers CD90, CD73, and CD105 (Fig. 1A–C). uASCs showed mostly negative staining for a cocktail of typical negative MSC markers, i.e. CD14/CD20/CD34/CD45 (Fig. 1F). The small population of positive cells in this case were thought to be CD34‐positive cells, as seen in Fig. 1D, which shows freshly isolated uASCs stained with an FITC‐labelled CD34‐specific antibody. A subpopulation of uASCs also stained for CD271, which is the low‐affinity NGF receptor p75 and a widely accepted MSC marker used for bone marrow‐derived stem cells (Fig. 1E); indeed 12.39% ± 1.22% of the total cell population was positive for CD271 (*n* = 4).

The trilineage differentiation potential of uASCs was examined, and it was found that they were capable of differentiation into all three lineages, demonstrating their *in vitro* multipotency (Fig. 1G). Indeed, following the three different mesodermal differentiation protocols, ASCs were able to produce fat droplets (stained with Oil Red O) as a sign of adipogenic differentiation, proteoglycans (stained with Toluidine Blue) as a sign of chondrogenic differentiation, and calcium deposits (stained with Alizarin Red S) as a sign of osteogenic differentiation. Undifferentiated cells that underwent the same staining protocols as negative controls showed no staining (Fig. S1).

### Morphological and physiological changes following glial differentiation

**Figure 2:**
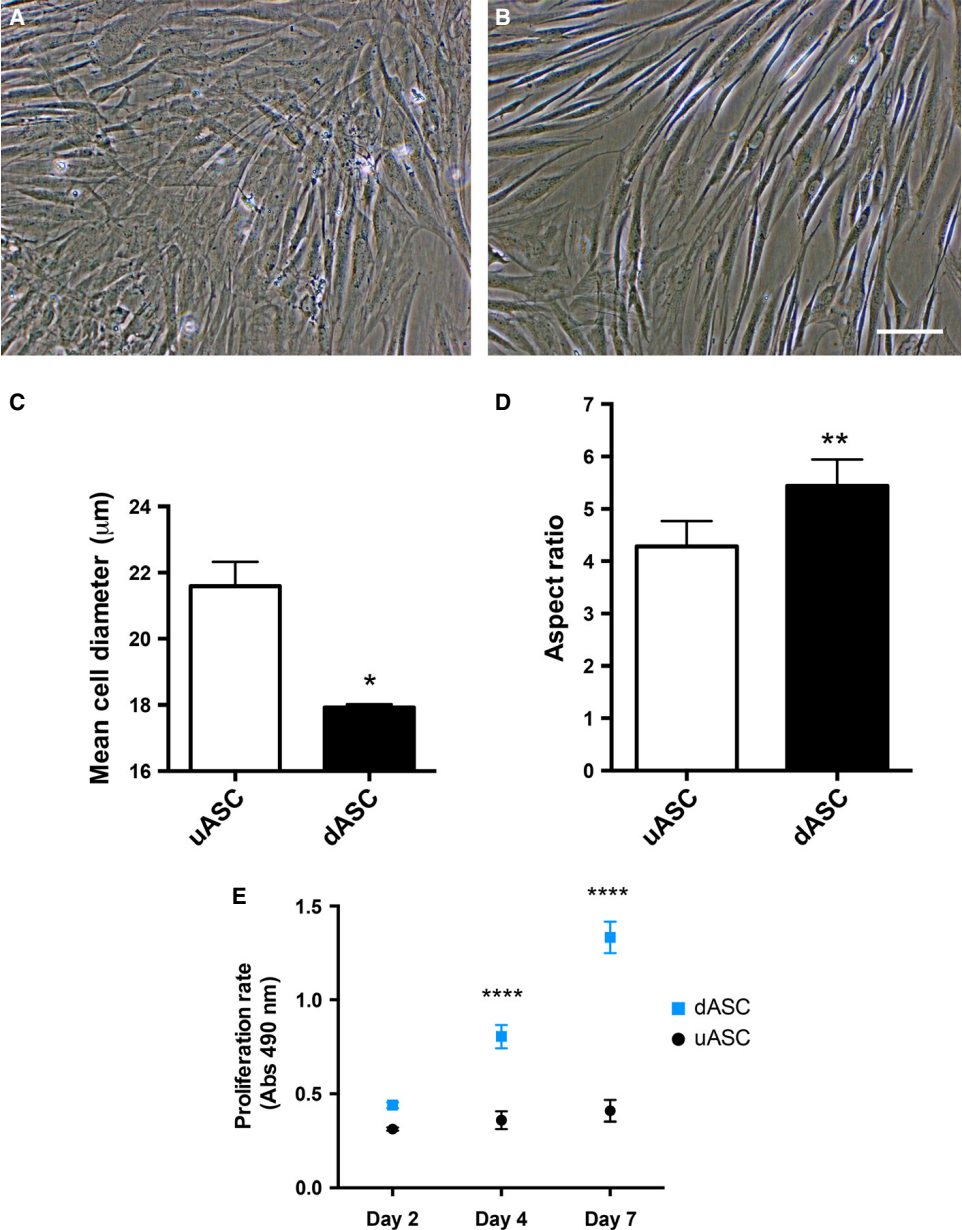
Morphological and physiological changes following glial differentiation. (A) uASCs show a fibroblast‐like morphology at postnatal days 1–2. (B) Upon differentiation, ASCs change to an elongated, SC‐like morphology. (C and D) dASCs show a reduced cell diameter (from 21.59 ± 0.73 μm to 17.93 ± 0.09 μm, *P < 0.05, *n* = 4) and an increased aspect ratio (from 4.28 ± 0.48 to 5.44 ± 0.50, **P < 0.01, *n* = 4) as compared with uASCs. (E) dASCs proliferate faster than uASCs, as determined with the MTS assay, at days 4 and 7 (****P < 0.0001, *n* = 4). Scale bar: 100 μm.

Prior to glial differentiation, human uASCs at passage 1–2 showed the flat, fibroblast‐like morphology typical of MSCs (Fig. 2A). Following the differentiation protocol described above, dASCs underwent a change towards an elongated, spindle‐shaped, SC‐like morphology (Fig. 2B). This change was observed starting from 3–5 days of growth factor exposure, and was maintained throughout the differentiation process and following repeated passaging. The mean cell diameter of dASCs exposed to the growth factors for 2 weeks was significantly lower than the uASC mean cell diameter (17.93 ± 0.09 μm vs. 21.59 ± 0.73 μm respectively, *P* < 0.05, *n* = 4; Fig. 2C). Glial differentiation also determined a significant increase in the aspect ratio in dASCs as compared with uASCs (5.44 ± 0.50 vs. 4.28 ± 0.48 respectively, *P* < 0.01, *n* = 4; Fig. 2D). Finally, following glial differentiation, dASCs showed a significantly higher proliferation rate following 4 and 7 days of culture than uASCs (*P* < 0.0001, *n* = 4; Fig. 2E).

### Gene expression and protein level changes in dASCs

In order to confirm successful glial differentiation, qRT‐PCR was used to assess the changes in gene expression for several neuroglial markers and neurotrophic factors in dASCs and to compare them with uASC expression levels.

**Figure 3:**
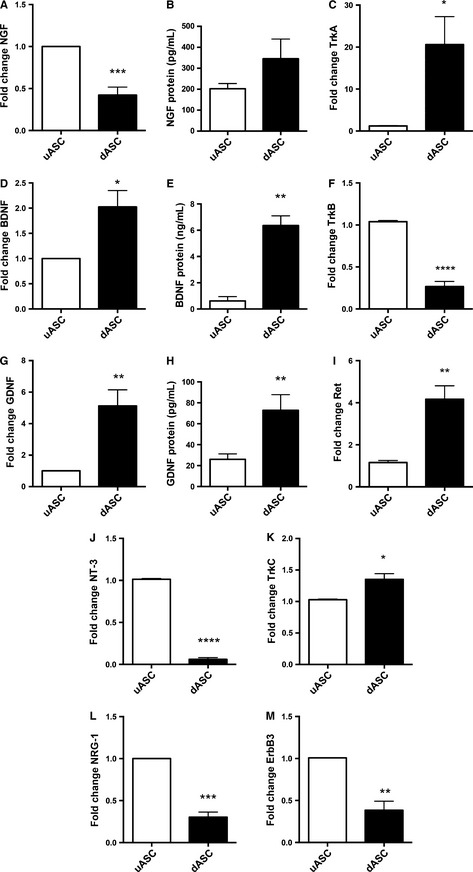
Gene expression and protein level changes of neurotrophic factors and receptors in dASCs: results of qRT‐PCR and ELISA on dASCs and uASCs. (A) NGF gene expression levels were decreased in dASCs as compared with uASCs (fold change of 0.42 ± 0.09, ***P < 0.001, *n* = 4). (B) NGF protein levels were increased, although the difference was not statistically significant (fold change of 345.2 ± 94.24 pg/mL in dASCs vs. 202.0 ± 24.94 pg/mL in uASCs, *P* = 0.2, *n* = 4). (C) TrkA receptor gene expression levels were strongly increased in dASCs (fold change of 20.62 ± 6.646, *P < 0.05, *n* = 4). (D) BDNF gene expression levels were higher in dASCs than in uASCs (fold change of 2.025 ± 0.3246, *P < 0.05, *n* = 4). (E) BDNF protein levels were higher in dASCs than in uASCs (6.36 ± 0.74 ng/mL and 0.62 ± 0.33 ng/mL, respectively, **P < 0.01, *n* = 4). (F) TrkB gene expression levels were significantly decreased in dASCs (fold change of 0.2665 ± 0.06155 vs. uASCs, ****P < 0.0001, *n* = 4). (G) GDNF gene expression levels were higher in dASCs than in uASCs (fold change of 5.128 ± 1.019, **P < 0.01, *n* = 4). (H) GDNF protein levels were higher in dASCs than in uASCs (72.88 ± 14.94 ng/mL and 26.02 ± 5.21 ng/mL, respectively, *P < 0.05, *n* = 4). (I) Ret gene expression levels were increased in dASCs (fold increase of 4.167 ± 0.6396 vs. uASCs, **P < 0.01, *n* = 4). (J) NT‐3 expression was almost absent in dASCs as compared with uASCs (****P < 0.0001, *n* = 4). (K) TrkC gene expression levels were increased in dASCs (fold change of 1.352 ± 0.09027 vs. uASCs, *P < 0.05, *n* = 4). (L and M) Expression levels of NRG‐1 and its receptor ErbB‐3 were decreased in dASCs as compared with uASCs [fold changes of 0.30 ± 0.06 (***P < 0.001) and 0.38 ± 0.10 (**P < 0.01), respectively, *n* = 4].

NGF gene expression levels were found to be significantly reduced following glial differentiation (0.42 ± 0.09 vs. uASCs, *P* < 0.001, *n* = 4; Fig. 3A). Nevertheless, protein levels were increased following differentiation (345.2 ± 94.24 pg/mL in dASCs vs. 202.0 ± 24.94 pg/mL in uASCs), although this difference did not reach statistical significance (*P* = 0.21, *n* = 4; Fig. 3B). Similarly, gene expression levels of tropomyosin receptor kinase A (TrkA), a high‐affinity receptor for NGF, were strongly increased in dASCs (fold change of 20.62 ± 6.646 vs. uASCs, *P* < 0.05, *n* = 4; Fig. 3C).

BDNF and GDNF gene expression levels were significantly increased in dASCs as compared with uASCs, with fold changes of 2.025 ± 0.3246 (Fig. 3D; *P* < 0.05, *n* = 4) and 5.128 ± 1.019 (Fig. 3G; *P* < 0.01, *n* = 4), respectively. These findings were also confirmed at the protein level with ELISA for measurement of the levels of secreted neurotrophins. Indeed, BDNF levels were significantly increased in dASCs (6.36 ± 0.74 ng/mL) as compared with uASCs (0.62 ± 0.33 ng/mL, *P* < 0.01, *n* = 4; Fig. 3E). Similarly, GDNF levels were significantly increased from 26.02 ± 5.22 pg/mL in uASCs to 72.88 ± 14.94 pg/mL in dASCs (*P* < 0.05, *n* = 4; Fig. 3H). Gene expression levels of tropomyosin receptor kinase B (TrkB), a tyrosine receptor kinase with high affinity for BDNF, and to a lesser extent neurotrophin‐3 (NT‐3), were significantly decreased in dASCs, with a fold change of 0.2665 ± 0.06155 as compared with uASCs (Fig. 3F; *P* < 0.0001, *n* = 4). In contrast, the GDNF receptor Ret was significantly upregulated following glial differentiation, with a fold change of 4.167 ± 0.6396 as compared with uASCs (Fig. 3I; *P* < 0.01, *n* = 4). Gene expression of NT‐3 was almost completely abolished following glial differentiation (Fig. 3J; *P* < 0.0001, *n* = 4); conversely, expression of its main receptor, tropomyosin receptor kinase C (TrkC) was slightly but significantly increased (fold change of 1.352 ± 0.09027 vs. uASCs, *P* < 0.05, *n* = 4; Fig. 3K).

Expression levels of neuregulin type 1 (NRG‐1) and its receptor tyrosine protein kinase epidermal growth factor receptor 3 (ErbB‐3) were significantly decreased in dASCs as compared with uASCs, with fold changes of 0.30 ± 0.06 (*P* < 0.001) and 0.38 ± 0.10 (*P* < 0.01), respectively (Fig. 3L and M, n = 4).

**Figure 4:**
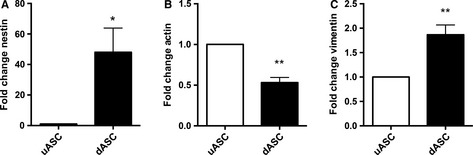
Gene expression level changes of intermediate filament and cytoskeleton proteins in dASCs. (A) Nestin gene expression levels were higher in dASCs than in uASCs (fold change of 48.05 ± 15.86, *P < 0.05, *n* = 4). (B) β‐Actin gene expression levels were significantly decreased in dASCs as compared with uASCs (fold change of 0.5321 ± 0.06254, *n* = 4, **P < 0.01). (C) Vimentin gene expression levels were significantly increased following differentiation (fold increase of 1.867 ± 0.1993 vs. uASCs, *n* = 4, **P < 0.01).

The neuronal precursor marker and intermediate filament protein nestin was substantially upregulated in dASCs (fold change of 48.05 ± 15.86 vs. uASCs, *P* < 0.05, *n* = 4; Fig. 4A). β‐Actin, one of the major non‐muscle cytoskeletal actins involved in maintaining cell structure and integrity, was significantly downregulated in dASCs as compared with uASCs (fold change of 0.5321 ± 0.06254, *n* = 4, *P* < 0.01; Fig. 4B). Finally, vimentin, a mesenchymal intermediate filament protein, was significantly upregulated following differentiation (fold increase of 1.867 ± 0.1993 vs. uASCs, *n* = 4, *P* < 0.01; Fig. 4C).

### Withdrawal of differentiation medium causes reversion of the dASC phenotype

**Figure 5:**
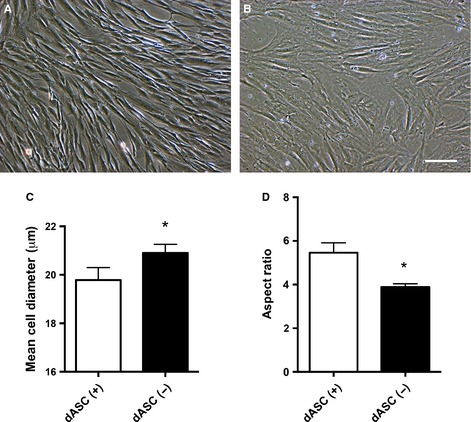
Withdrawal of differentiation medium causes reversion of dASC morphology. (A and B) dASCs maintained in differentiation medium [A: dASCs(+), αMEM plus growth factors and forskolin] were compared with dASCs that had been removed from differentiation medium for 72 h [B: dASC(−), αMEM with no growth factors or forskolin], and this showed that dASCs(−) rapidly lose their SC‐like phenotype. (C) The mean cell diameter was increased in dASCs(−) as compared with dASCs(+), from 19.79 ± 0.51 μm to 20.90 ± 0.35 μm (*P < 0.05, *n* = 4). (D) The aspect ratio was significantly decreased in dASCs(−) as compared with dASCs(+), from 5.459 ± 0.4541 to 3.895 ± 0.1419 (*P < 0.05, *n* = 4). Scale bar: 100 μm.

In order to assess the effect of withdrawal of the differentiation medium on dASCs, observations were made on dASCs following removal of growth factors for 72 h [dASCs(−)] as compared with dASCs maintained in differentiation medium [dASs(+)]. Interestingly, dASC morphology quickly reverted in the absence of the differentiation medium, returning from the spindle‐shaped dASC(+) morphology acquired during the 18‐day differentiation protocol (Fig. 5A) to the initial fibroblast‐like dASC(−) morphology (Fig. 5B). The removal of growth factors also caused reversion of other morphological parameters, such as the mean cell diameter, which changed from 19.79 ± 0.51 μm in dASCs(+) to 20.90 ± 0.35 μm in dASCs(−) (Fig. 5C; *P* < 0.05, *n* = 4), and the aspect ratio, which changed from 5.459 ± 0.4541 μm in dASCs(+) to 3.895 ± 0.1419 μm in dASCs(−) (Fig. 5D; *P* < 0.05, *n* = 4).

**Figure 6:**
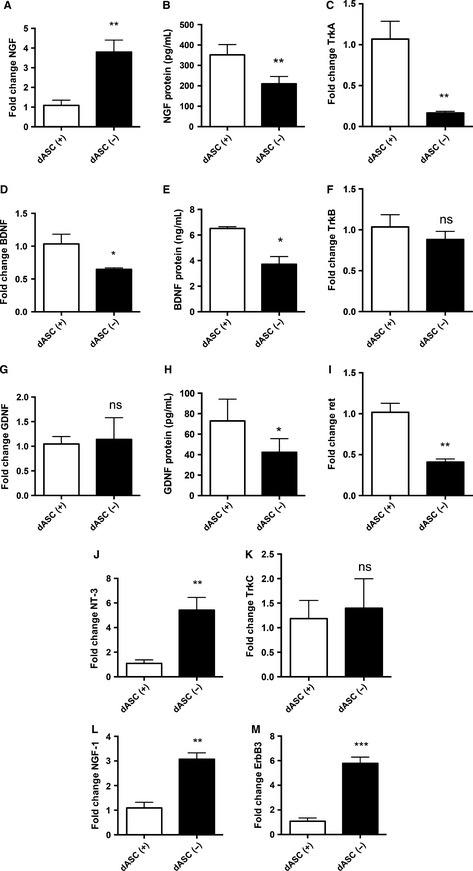
Withdrawal of differentiation medium causes reversion of the dASC phenotype (neurotrophic factors and receptors). Expression levels in dASCs withdrawn from differentiation medium [dASCs(−), αMEM with no growth factors or forskolin] and dASCs maintained in differentiation medium [dASCs(+), αMEM plus growth factors and forskolin] were investigated with qRT‐PCR and ELISA. (A and B) NGF gene expression levels were increased in dASCs(−) as compared with dASCs(+) (fold change of 3.80 ± 0.61, **P < 0.01, *n* = 4), but protein levels were decreased from 351.8 ± 50.27 pg/mL in dASCs(+) to 210.3 ± 35.69 pg/mL in dASCs(−) (**P < 0.01, *n* = 4). (C) TrkA gene expression levels were significantly decreased in dASCs(−) as compared with dASCs(+) (fold change of 0.1665 ± 0.02035, **P < 0.01, *n* = 4). (D and E) BDNF gene expression levels were decreased in dASCs(−) as compared with dASCs(+) (fold change of 0.65 ± 0.02, *P < 0.05, *n* = 4), and BDNF protein levels were also decreased in dASCs(−) as compared with dASCs(+) (6.51 ± 0.14 ng/mL vs. 3.72 ± 0.61 ng/mL, *P < 0.05, *n* = 4). (F) TrkB gene expression levels remained unchanged following withdrawal of stimulation. (G and H) GDNF gene expression remained unchanged between dASCs(−) and dASCs(+), but protein levels were significantly decreased in dASCs(−) as compared with dASCs(+) (42.25 ± 13.35 pg/mL vs. 73.00 ± 21.19 pg/mL, *P < 0.05, *n* = 4). (I) Ret gene expression levels were also significantly decreased in dASCs(−) [fold change of 0.4086 ± 0.03890 vs. dASCs(+), *n* = 4, **P < 0.01]. (J and K) NT‐3 gene expression levels were increased in dASCs(−) as compared with dASCs(+) (fold change of 5.423 ± 1.032, **P < 0.01, *n* = 4), but TrkC expression levels were unchanged. (L) ErbB‐3 gene expression levels were increased in dASCs(−) as compared with dASCs(+) (fold change of 5.78 ± 0.51, ***P < 0.001, *n* = 4). (M) NRG‐1 gene expression levels were increased in dASCs(−) as compared with dASCs(+) (fold change of 3.07 ± 0.26, **P < 0.01, *n* = 4).

The gene and protein expression levels of neurotrophins and other neuroglial markers were also assessed following growth factor withdrawal. NGF gene expression levels were significantly increased in dASCs(−) (fold change of 3.80 ± 0.61, *P* < 0.01, *n* = 4; Fig. 6A). Conversely, levels of secreted NGF were significantly decreased in dASCs(−) (210.3 ± 35.69 pg/mL) as compared with dASCs(+) (351.8 ± 50.27 pg/mL, *P* < 0.01, *n* = 4; Fig. 6B). This was accompanied by decreases in the expression levels of the NGF receptor TrkA in dASCs(−) as compared with dASCs(+) (fold change of 0.1665 ± 0.02035, *n* = 4, *P* < 0.01; Fig. 6C).

BDNF gene expression levels were significantly decreased after withdrawal [fold change of 0.65 ± 0.02 vs. dASCs(+), *P* < 0.05, *n* = 4; Fig. 6D] This effect was replicated at the protein level, as BDNF levels were significantly decreased after 72 h of growth factor withdrawal (6.51 ± 0.14 ng/mL vs. 3.72 ± 0.61 ng/mL, *P* < 0.05, *n* = 4; Fig. 6E). Interestingly, levels of the BDNF receptor TrkB did not change following the withdrawal of the growth factors (Fig. 6F). Similarly, levels of GDNF remained unchanged between dASCs(+) and dASCs(−) at the gene level (Fig. 6G); however, GDNF protein secretion was significantly decreased following withdrawal [73.00 ± 21.19 pg/mL in dASCs(+) vs. 42.25 ± 13.35 pg/mL in dASCs(−), *n* = 4, *P* < 0.05; Fig. 6H]. Gene expression levels of the Ret receptor were also significantly decreased in dASCs(−), with a fold change of 0.4086 ± 0.03890 vs. dASCs(+) (*n* = 4, *P* < 0.01; Fig. 6K).

NT‐3 expression levels were significantly increased upon withdrawal [fold change of 5.423 ± 1.032 vs. dASCs(+), *n* = 4, *P* < 0.01; Fig. 6J], whereas expression levels of its receptor TrkC were unchanged (Fig. 6K). Gene expression levels of both NRG‐1 and its receptor ErbB‐3 were significantly increased in dASCs(−) (fold change of 3.07 ± 0.26 for NRG‐1, *n* = 4, *P* < 0.01; fold change of 5.78 ± 0.51 for ErbB‐3, *n* = 4, *P* < 0.001; Fig. 6L and M).

**Figure 7:**
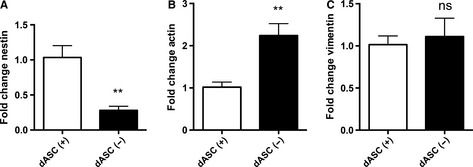
Withdrawal of differentiation medium causes reversion of the dASC phenotype (intermediate filaments and cytoskeleton). (A) Nestin gene expression levels were reduced in dASCs(−) as compared with dASCs(+) (fold change of 0.28 ± 0.06, **P < 0.01, *n* = 4). (B) β‐Actin gene expression levels were increased in dASCs(−) [fold change of 2.239 ± 0.2841 vs. dASCs(+), **P < 0.01, *n* = 4). (C) Vimentin expression levels did not change.

Nestin expression levels were significantly decreased in dASCs(−) [fold change of 0.28 ± 0.06 vs. dASCs(+), *n* = 4, *P* < 0.01; Fig. 7A]. β‐Actin gene expression levels were increased following growth factor withdrawal [fold increase of 2.239 ± 0.2841 vs. dASCs(+), *P* < 0.01, *n* = 4; Fig. 7B], whereas vimentin expression levels did not change (Fig. 7C).

## Discussion

This study highlights a potential problem for the proposed clinical use of differentiated human ASCs. Following the Dezawa differentiation protocol over a period of 18 days, we have demonstrated the change in phenotype of human ASCs to an SC‐like morphology, with greatly increased gene and protein expression levels for the growth factors BDNF and GDNF. However, after removal of the induction factors from the medium, the same cells very rapidly underwent reversion of their phenotype to stem cell morphology, and significantly reduced their growth factor expression. All results are summarized in Table 2.

**Table 2:** Summary of results: changes in gene expression, protein levels and cell morphology after differentiation (as compared with undifferentiated cells) and after withdrawal from differentiation medium (as compared with cells maintained in differentiation medium)

	Differentiation	Withdrawal
Morphology		
Cell diameter	↓	↑
Aspect ratio	↑	↓
Neurotrophins		
*NGF*	↓	↑
NGF protein	↑	↓
*BDNF*	↑	↓
BDNF protein	↑	↓
*GDNF*	↑	‐
GDNF protein	↑	↓
*NT‐3*	↓↓	↑
Neurotrophin receptors		
*TrkA*	↑↑	↓
*TrkB*	↓	–
*TrkC*	↑	–
*Ret*	↑	↓
Neuregulin system		
*NRG‐1*	↓	↑
*ErbB3*	↓	↑
Cytoskeleton		
*Nestin*	↑↑	↓
*Actin*	↓	↑
*Vimentin*	↑	–

One arrow indicates a change <10‐fold in magnitude; two arrows indicate a change >10‐fold in magnitude. Dashes indicate no significant change.

The Dezawa differentiation protocol was first found to induce differentiation towards SCs by the use of rat bone marrow MSCs (Dezawa *et al*., 2001). It has subsequently been used to induce differentiation in rat and human ASCs for many *in vitro* and *in vivo* models of peripheral nerve regeneration (Kuroda *et al*., 2011; Faroni *et al*., 2013b). Although *in vitro* models have shown consistent promise in measuring the impact of the differentiated vs. the undifferentiated stem cells, there is no report of a control study showing that the differentiating medium is required to maintain that phenotype. As a consequence, *in vivo* models comparing the treated and non‐treated stem cells are seemingly less effective. Indeed, the disclosure that the differentiated cells may revert to a stem cell phenotype when removed from their permissive environment is rarely acknowledged.

Our findings demonstrate that differentiation of human ASCs leads to increased expression of GDNF and BDNF and reduced expression of NT‐3, in keeping with the findings of other authors (Tomita *et al*., 2013; Kingham *et al*., 2014). However, this could be attributable to growth factor stimulation, in particular by GGF‐2, rather than being a sign of differentiation. Indeed, GGF‐2 is one of the main axonal signals that maintains the SCs *in vivo*, and, reciprocally, SCs produce BDNF and GDNF for neuron maintenance (Topilko *et al*., 1996; Terenghi, 1999). In our *in vitro* differentiation model, we provide high levels of GGF‐2, which probably mimics the paracrine signals observed *in vivo*, prompting dASCs to produce more BDNF and GDNF.

Conversely, NT‐3 levels are reduced following glia differentiation. This is not surprising, as NT‐3 is only expressed following injury in SCs, where the contact with the axons is missing (Meier *et al*., 1999). Consistently, in our model the presence of GGF‐2 reduces NT‐3 expression levels, which are re‐established when the growth factors are removed, possibly mimicking the nerve injury situation.

In this study, we consistently found reduced gene expression of NGF, which is in contrast to previous reports on rat and human dASCs (Kingham *et al*., 2007, 2014; Tomita *et al*., 2013). NGF expression has been widely reported to be biphasic in SCs *in vitro* (Matsuoka *et al*., 1991; Zafra *et al*., 1991), and *in vivo* following injury (Meyer *et al*., 1992). This implies that the time of collection following cell stimulation is critical, and that perhaps gene expression analysis at different time points is required. The discrepancy may also be attributable to the different glial induction signals, because, in this study, we used GGF‐2, which is an NRG‐1 type II isotype, whereas in the other studies the commercially available NRG‐1 type I isotype was used. Furthermore, our assessment of the NGF levels was carried out with qRT‐PCR, whereas other authors used qualitative reverse transcriptase polymerase chain reaction, which is another possible reason for the discrepancy (Kingham *et al*., 2014).

In contrast to gene expression levels, the level of secreted NGF protein was increased, although the difference did not reach statistical significance. Other authors, who have found similar levels of patient variability, have reported this pattern of NGF protein levels following differentiation (Tomita *et al*., 2013; Kingham *et al*., 2014).

Following withdrawal of induction medium, BDNF gene and protein expression levels were significantly decreased, whereas GDNF gene expression remained similar, with protein expression being significantly reduced. It is of interest that NGF gene and protein expression levels respond differentially, which is a consistent trend observed in both the differentiation and withdrawal protocols. This differential response between mRNA and protein is not unusual (Greenbaum *et al*., 2003), and, in the case of NGF, it could be attributable to mechanisms related to RNA stabilization, and NGF metabolic kinetics, degradation, and recycling (Matsuoka *et al*., 1991; Meyer *et al*., 1992).

It is to be expected that the changes in expression in response to stimulation medium and withdrawal are not restricted to the neurotrophins, but also apply to their receptors. We have studied the Trk family of receptor kinases (TrkA, TrkB, and TrKC) and the Ret tyrosine kinase. Although the neurotrophins bind preferentially to specific receptors (NGF to TrkA, BDNF to TrkB, NT‐3 to TrkC, and GDNF to Ret), these pairings are not absolute; for example, NT‐3 can also bind to TrkA or TrkB (Bibel & Barde, 2000). We found that receptor expression was upregulated following glial differentiation, with the exception of TrkB. Upon withdrawal, TrkA and Ret expression levels reverted, whereas TrkB and TrkC expression levels were maintained at the levels of differentiated cells. The neurotrophin and tyrosine kinase receptor signalling pathways are complex, often interconnected, and strictly related to SC pathophysiology in terms of migration, survival, differentiation, and response to injury (Yamauchi *et al*., 2003; Pitts *et al*., 2006; Richner *et al*., 2014). For this reason, gene expression may not follow an exclusive and specific receptor–ligand pattern, owing to cross‐binding and other compensatory mechanisms.

NRG‐1 and its receptor ErbB‐3 showed increased gene expression levels following withdrawal of growth factors, and this contrasts with the downregulating response during differentiation. The neuregulin signalling system is pivotal for SC development and survival, and is fundamental for the paracrine–autocrine neuroglia communication during development and repair (Carroll *et al*., 1997; Stassart *et al*., 2013). This system strictly correlates with the expression of neurotrophic factors from developing or injured SCs (Ma *et al*., 2011). Indeed, axon‐derived neuregulins provide support to SCs, and, when these signals are interrupted following injury, SCs respond by changing phenotype and upregulating several growth factors, which, in turn, signal back to the neurons to promote regeneration (Reynolds & Woolf, 1993; Snider *et al*., 2002; Makwana & Raivich, 2005). The NRG‐1 gene is the best characterized gene among the neuregulin gene family (Taveggia *et al*., 2010). This complex gene encodes multiple isoforms derived from alternative splicing or from the use of different promoters, which are fundamental for several mechanisms of SC development, myelination, nerve response to injury, and nerve regeneration (Gambarotta *et al*., 2013).

We believe that, with our *in vitro* differentiation model, we are reproducing the *in vivo* paracrine signalling between SCs (dASCs) and neurons (GGF‐2), which determines the changes in the expression of neurotrophic factors and their receptors. Conversely, upon withdrawal of these signals, we reproduce the nerve injury situation, with unbalanced artificial neuroglia signalling and subsequent changes in growth factor expression. This could also explain the increase in NGF expression following withdrawal, in line with the increase in NGF expression following injury *in vivo* (Terenghi, 1999).

The expression levels of cytoskeletal components also show a differential response to induction medium and withdrawal. Nestin is an intermediate filament protein expressed in SC precursors but not in other glial cell types (Friedman *et al*., 1990; Woodhoo *et al*., 2007). During development, it is expressed in proliferating neuroglial progenitors; it is downregulated upon differentiation and adulthood, although it can be transiently expressed following injury, and it is present in cell subpopulations of many tissues (Michalczyk & Ziman, 2005). Nestin is widely accepted as a marker to identify stem cell precursors that are able to generate SC‐like cells from several different stem cell niches, including fat (Amoh *et al*., 2012; Faroni *et al*., 2012; Hauser *et al*., 2012; Mii *et al*., 2013; Martens *et al*., 2014). However, it should not be considered to be a marker of differentiated SCs. In this study, we observed a significant increase in nestin expression, which was reversed upon stimulation withdrawal. It is possible that our differentiation protocol can produce partial stem cell differentiation to the state of SC precursors, but not full SC maturation. In contrast, actin expression is downregulated in the presence of differentiation medium, and returns to baseline following withdrawal. The rearrangement of the actin cytoskeleton is critical to the multiple functions of SC and actin is expressed highly in SCs that are fully differentiated to a myelinating spindle‐shaped phenotype (Li *et al*., 2003; Procacci *et al*., 2012). Although this is not observed in our spindle‐shaped dASCs, it may be that protein is differentially expressed or, alternatively, that another cytoskeletal component facilitates the change in morphology (Jessen & Mirsky, 2005). Vimentin is an intermediate filament protein that is expressed exclusively in myelin‐forming SCs and upregulated following nerve injury (Neuberger & Cornbrooks, 1989; Jessen & Mirsky, 1991). Here, we observed a two‐fold increase in vimentin gene expression in response to differentiation medium, which was maintained following withdrawal. Of all of the cytoskeletal components investigated, nestin may have the most influence on the changes in morphology; however, further work will be required to investigate the effect of eliminating its expression on dASCs on the development of a spindle‐shaped morphology.

Defining a particular and unique SC phenotype is exceptionally challenging, owing to the great plasticity of SCs, starting from development and extending into adulthood and following injury. Many studies have addressed SC gene expression changes in development and following injury, and have identified numerous genes that are expressed in the different stages, often with great overlap (Buchstaller *et al*., 2004; D'Antonio *et al*., 2006; Woodhoo & Sommer, 2008). Nevertheless, growth factors such as NGF, BDNF, GDNF, and NT‐3, together with their receptors, are widely recognized as key players in SC physiology, development, and response to injury (Terenghi, 1999), and in this study we have shown dramatic changes in the expression of these important markers following glial differentiation, and after withdrawal of glial stimulation.

Several different protocols have been employed to obtain SCs from induced pluripotent stem cells, and most of them require several weeks of *in vitro* differentiation (Wang *et al*., 2011; Ma *et al*., 2015). It is also important to note that the protocol that we employed was originally developed and optimized for rodent cultures. However, rat development is much shorter than human development, so longer differentiation protocols might be needed to achieve complete differentiation. Indeed, studies based on rodent cultures often fail to translate to human cell‐based studies, owing to great interspecies differences (Davidson *et al*., 2014; Dib‐Hajj, 2014). In the case of SCs, this is further aggravated by the fact that most of the studies conducted on rat SCs have been performed mostly with neonatal cultures (Brockes *et al*., 1979), whereas human SCs are normally obtained from adult patient nerve biopsies, which are burdened by a slower growth rate and require optimized protocols for harvesting and expansion (Morrissey *et al*., 1991; Casella *et al*., 1996). In‐depth whole genome analysis of the differences/similarities between human uASCs and dASCs in comparison with native human SCs could provide further information on the differentiation state of the stimulated cells.

Although our experimental model cannot precisely replicate the hostile environment that clinically transplanted cells would experience in a peripheral nerve gap injury, we have demonstrated the impact of removing SC‐induction factors from the stem cells *in vitro*. This would be one feature of the stem cell transplant process, although other factors, such as the inflammation and hypoxic milieu, are certain to influence the action of the transplanted cells, and it would seem unlikely these would offer as permissive an environment as the SC induction medium. The implications for the phenotype of transplanted dASCs will need to be tested *in vivo* to better model the influence of the clinical environment.

Our results indicate that caution has to be used in defining the term ‘differentiation’ as applied to past protocols for human ASCs. Furthermore, this study suggests that an alternative route for the clinical implementation of stem cell therapy in peripheral nerve injury might be considered. It is clear that uASCs can improve nerve regeneration outcomes, and even reduce sensory neuronal cell death (Reid *et al*., 2011; Kingham *et al*., 2014), so it may be a more direct route to clinical translation. Further refinements to cell therapy may be approached by the use of an alternative protocol providing true differentiation of the stem cells, perhaps by sustained delivery of growth factors to the cells, including during the post‐transplant stage, or alternatively by identifying neurotrophic subpopulations of the heterogeneous uASC population that are able to generate more stable SC‐like cells (Quirici *et al*., 2010; Johal *et al*., 2015).

## Supporting information

Fig. S1. Multipotency staining controls. uASC cultures did not show any staining with Oil Red O (A), Toluidine Blue (B) and Alizarin Red S (C), confirming the specificity of the stains used to assess differentiation in adipocytes, chondrocytes and osteocytes in Fig. 1G.
