# Supplementary figures and images for: Human Schwann‐like cells derived from adipose‐derived mesenchymal stem cells rapidly de‐differentiate in the absence of stimulating medium
Source: Eur J Neurosci. 2015 Sep 18;43(3):417–30. doi: 10.1111/ejn.13055 (PMC4744694; doi:10.1111/ejn.13055)

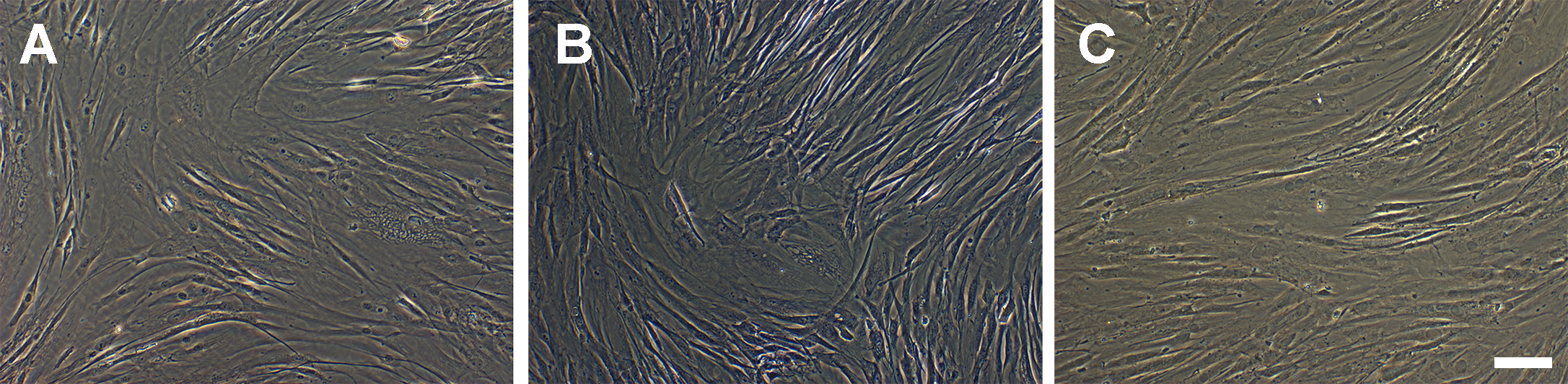

Supplement: Supplementary file 1 — Fig. S1. Multipotency staining controls. uASC cultures did not show any staining with Oil Red O (A), Toluidine Blue (B) and Alizarin Red S (C), confirming the specificity of the stains used to assess differentiation in adipocytes, chondrocytes and osteocytes in Fig. 1G. [file EJN-43-417-s001.tif]
